# Two cases in which 3D MRI was used to differentiate between a disc mass that mimics a tumor and neurinoma

**DOI:** 10.1186/s12891-018-2070-2

**Published:** 2018-05-22

**Authors:** Jingyu Jia, Qiangqiang Wei, Tianlong Wu, Dingwen He, Xigao Cheng

**Affiliations:** 1 Department of Orthopaedics, The Second Affiliated Hospital of Nanchang University, Nanchang City, 330006 Jiangxi Province China; 2 Department of Orthopaedics, The Second Affiliated Hospital of Nanchang University, Nanchang City, Jiangxi Province China

**Keywords:** Lumbar disc herniation, 3D fast-field echo with water selective excitation, Disc sequestration, Mimicking tumor

## Abstract

**Background:**

Since disc sequestration that mimics a tumor is rare and sometimes presents with an atypical appearance upon magnetic resonance imaging (MRI), it is often confused with other more common epidural and intradural neoplasms, particularly neurinoma. Open surgery is necessary due to the difficult of achieving a definitive diagnosis using computed tomography, MRI, and gadolinium- enhanced MRI prior to operation. Herein, we describe the use of coronal MR images of 3D fast-field echo with water selective excitation in the diagnosis of disc sequestration mimicking a tumor.

**Case presentation:**

Two patients were admitted to our hospital with back pain, radiating pain, and hypoesthesia in the right lower limb. MRI revealed tumor-like masses in the lateral recess of L3 and posterior to the body of L4. The initial diagnosis indicated disc sequestration mimicking a tumor and neurinoma. The coronal MR images of 3D fast-field echo with water selective excitation showed a clear boundary between the tumor-like mass and the nerve root. Moreover, the mass was also completely separated from the dura. Therefore, neurinoma was excluded as a possible diagnosis prior to operation. Surgical excision to perform removal of the gross mass was performed in one patient. The histopathological diagnosis was consistent with the 3D fast-field echo with water-selective excitation MRI. Another patient was successfully treated by minimally invasive endoscopic surgery.

**Conclusions:**

Disc sequestration that mimics a tumor is difficult to diagnose preoperatively. As a non–invasive strategy, coronal MR images of 3D fast-field echo with water selective excitation is a helpful imaging tool for differentiating between diagnosis of disc sequestration that mimics a tumor and neurinoma prior to operation. If the disc fragment of mimicking tumor can be identified prior to operation, open surgery may not be necessary for all patients. Minimally invasive endoscopic surgery also is an alternative strategy.

## Background

Disc sequestration is defined as the migration of a herniated disc fragment into the epidural space such that it is completely separated from the parent disc [1]. Magnetic resonance imaging (MRI) is the first diagnostic tool and gold standard for evaluating spinal pathologies [2]. However, in rare cases, the appearance of disc sequestration upon MRI is similar to that of a common epidural tumor, such as neurinoma or meningioma [3–5]. In 1993, Emamian et al. [6] detailed one case of lumbar herniated disk mimicking a neurinoma. Since then, mimicking tumor discs have been reported in several case reports [1–5, 7–22]. Open surgery was performed in all cases [1–22] since the surgeons were unable to make a definitive diagnosis using computed tomography (CT), MRI, and gadolinium-enhanced (Gd) MRI before operation. Herein, the authors successfully identified the disc fragment that mimicked a neurinoma using the coronal MRI of 3D fast-field echo with water selective excitation prior to surgery (3D MRI).

## Case presentation

### Case 1

**Fig. 1:**
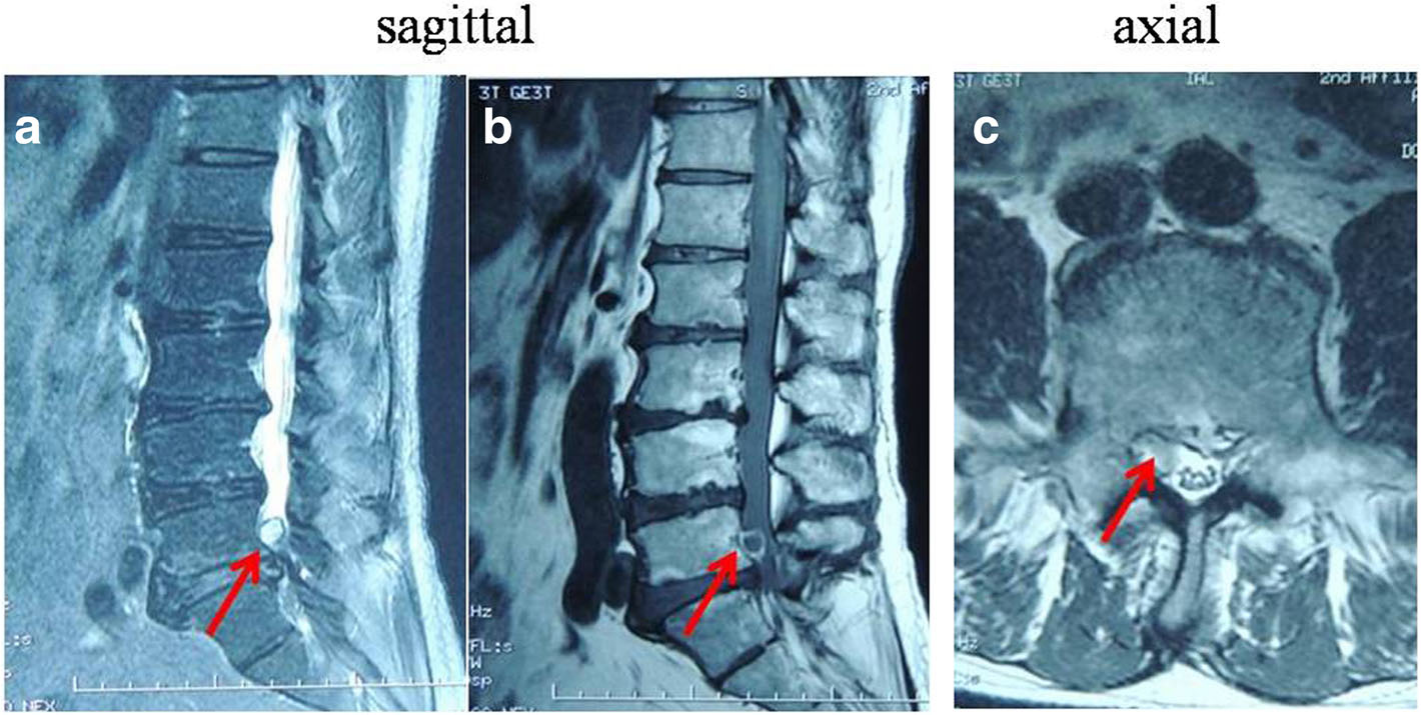
Preoperative MRI of the lumbar spine without intravenous contrast. T2-weighted MRI in the sagittal and axial planes showed a mass that mimicked a neurinoma posterior to the body of L4 (**a** and **c**). T1-weighted MRI in the sagittal planes showed a tumor-like mass with signal intensity (**b**). Meanwhile, the periphery of the mass was hyperintense and ring-like on T1WI (**b**)

**Fig. 2:**
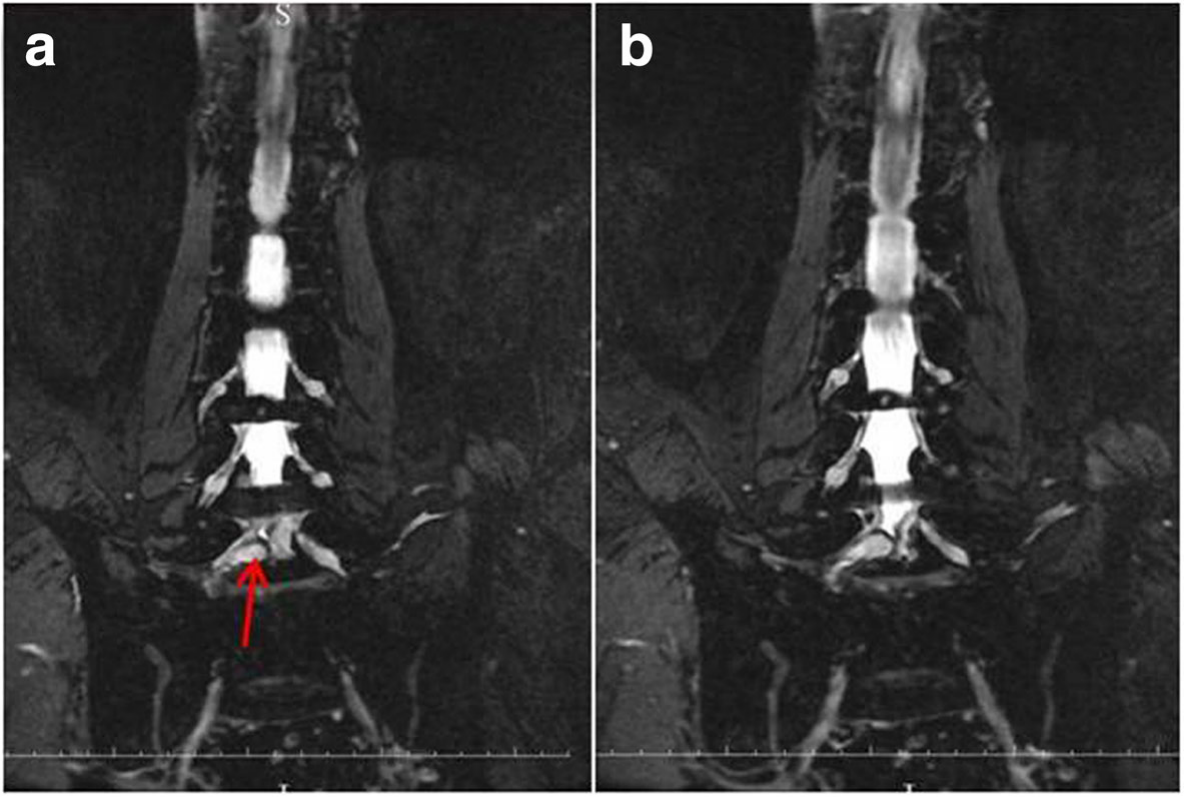
Coronal MR images of 3D fast-field echo with water selective excitation. Clear boundary was observed between the mass and nerve root by coronal MR images (**a**). Meanwhile, it was found that the mass was also separated with dura (**b**)

**Fig. 3:**
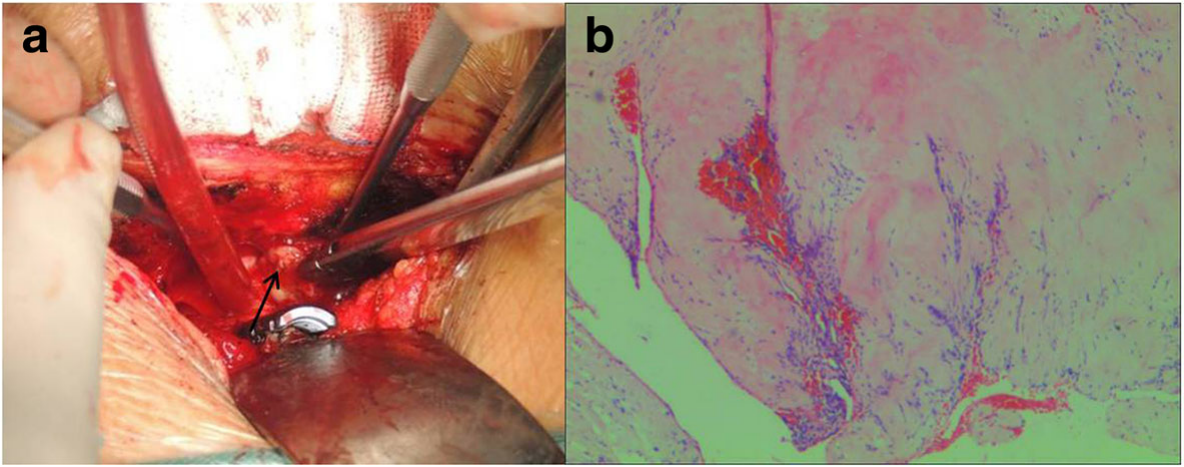
Intraoperative view found the mass of mimicking tumor (black arrow) was disc fragment (**a**). The histopathological diagnosis confirmed the intraoperative observation that the mass was a disc fragment (**b**). (hematoxylin and eosin × 40)

A 57-year-old man with a 10-year history of intermittent pain of the lower back and a 7-month history of intermittent pain of the right lower limb was admitted to our clinic. He complained of increased pain and hypoesthesia of the right lower limb that had started 1 month prior. Physical examination revealed that the pain was located in the distribution of L4 and weakness of the left extensor digitorum longus. Using a 10-cm visual analog scale (VAS), the patient reported the intensity of his leg pain to be 7/10, with an Oswestry disability index (ODI) score of 70. Negative Lasegue’s sign was shown bilaterally on the lower limbs. T1-weighted images (T1WI) of MRI revealed a tumor-like mass with signal intensity posterior to the body of L4 (Fig. 1b). But, the periphery of the mass demonstrated a hyperintense ring on T1WI (Fig. 1b). Similarly, the mass was hyperintense on T2-weighted images (Fig. 1a). We suspected neurinoma since the mass presented as a similar appearance as neurinoma on MRI (Fig. 1a–c). To further diagnose the mass, the coronal MRI of 3D fast-field echo with water selective excitation (3D MRI) was performed, which revealed a clear boundary between the mass and nerve root (Fig. 2). Meanwhile, the mass was also separated with dura (Fig. 2). Therefore, we excluded neurinoma as a possible diagnosis prior to operation. Surgical excision with gross removal of the mass was performed in our hospital (Fig. 3a). The histopathological diagnosis was consistent with a degenerated intervertebral disc (Fig. 3b). The patient’s symptoms improved immediately after surgery. He identified the strength of the lower extremities improving and recovered fully after 5 months.

### Case 2

**Fig. 4:**
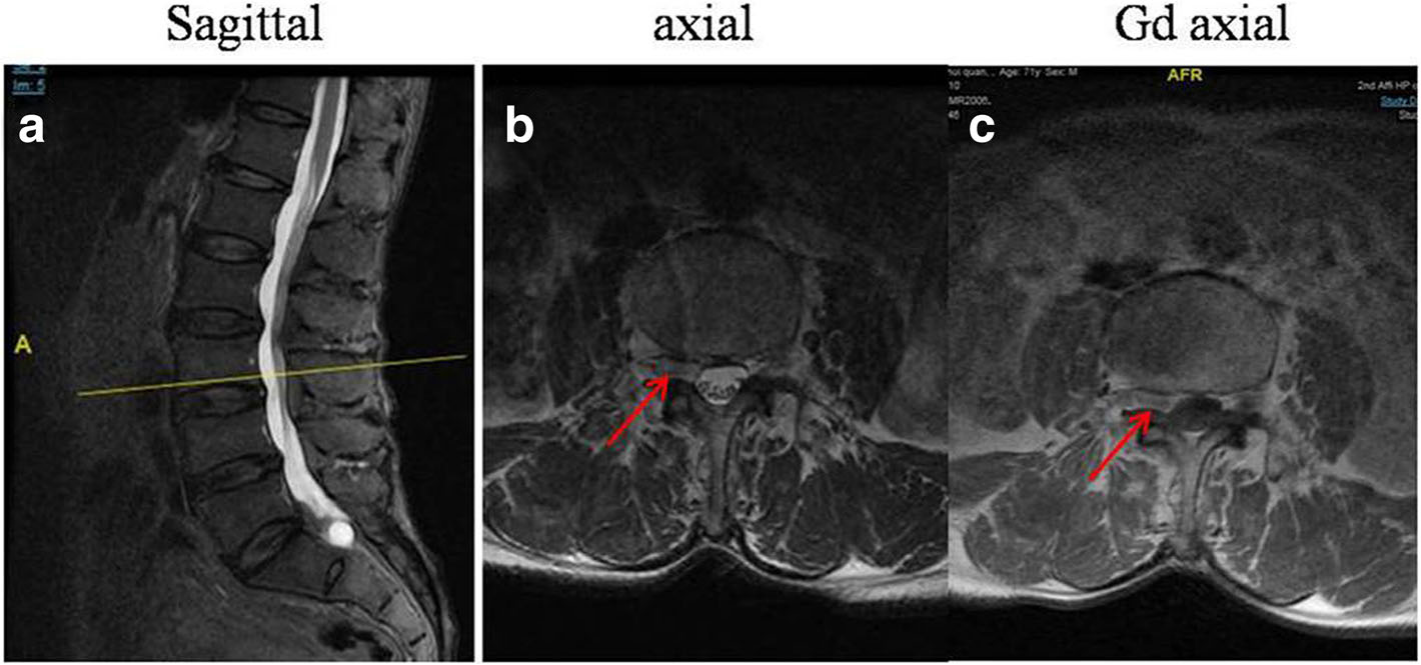
Preoperative MRI of the lumbar spine without intravenous contrast and with gadolinium- enhanced MRI. T2-weighted MRI in the sagittal plane was used to aid in localization (**a**). T2-weighted images in the axial planes revealed a dumbbell mass in the lateral recess (**b**). Gadolinium-enhanced MRI demonstrated a heterogeneous ring-like enhancement at the periphery of the mass (**c**)

**Fig. 5:**
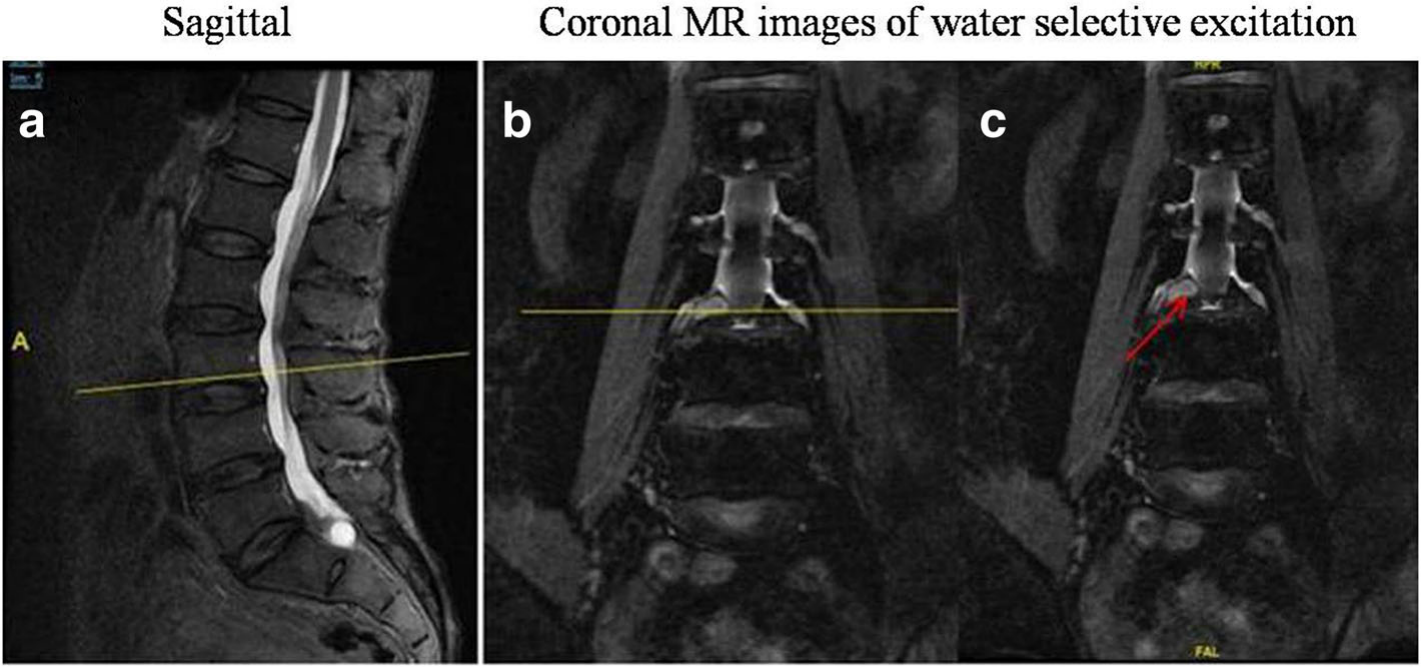
Coronal MR images of 3D fast-field echo with water selective excitation. T2-weighted MRI in the sagittal and axial plane was used to aid in localization (**a** and **b**). A clear boundary was observed between the mass and nerve root by coronal MRI (**c**)

**Fig. 6:**
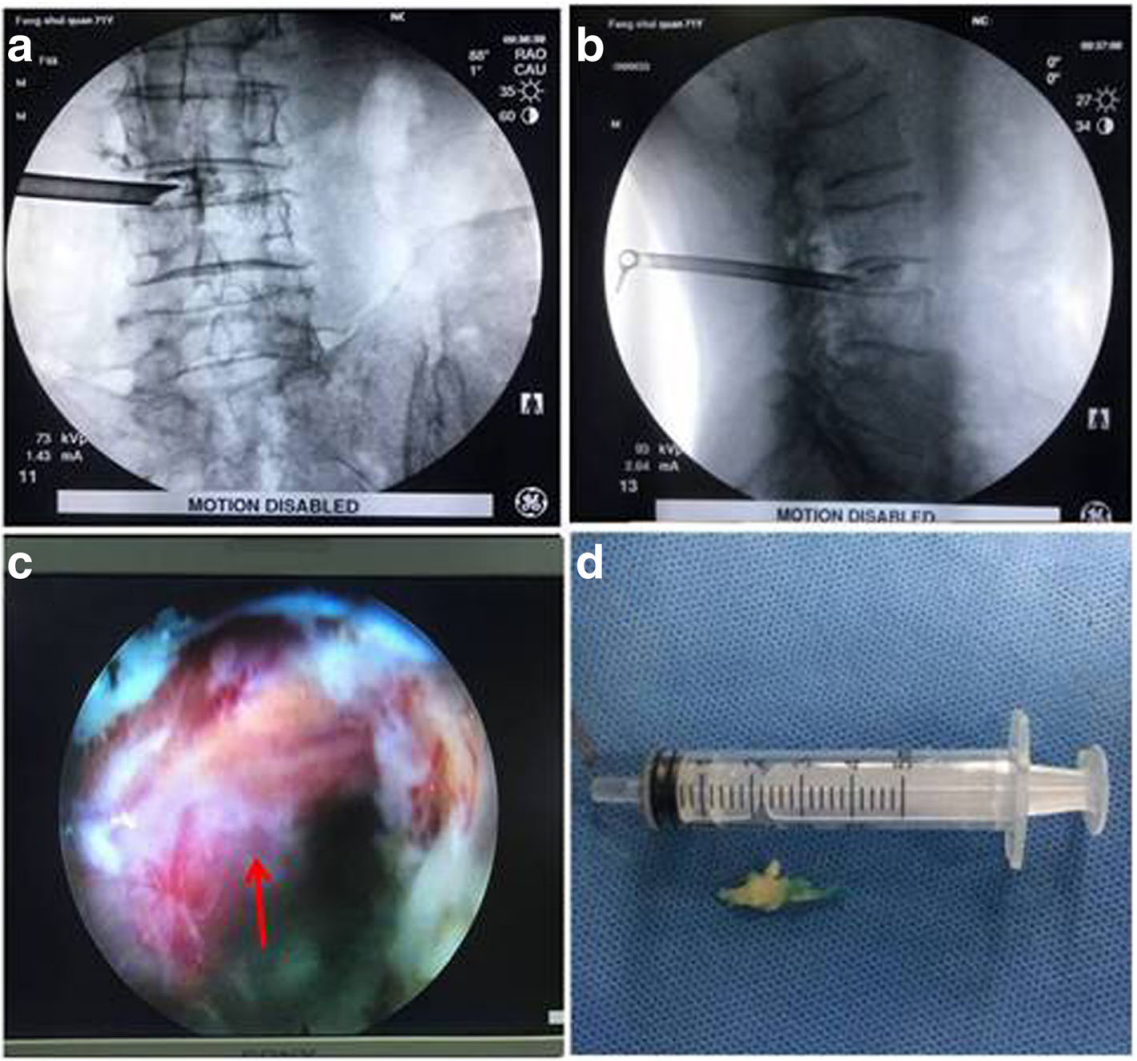
Intraoperative C-arm image showed the placement of the cannula on anteroposterior and lateral views (**a** and **b**). The exiting nerve root (red arrow) was exposed (**c**) and the mass (**d**) was removed under invasive endoscopic surgery

**Fig. 7:**
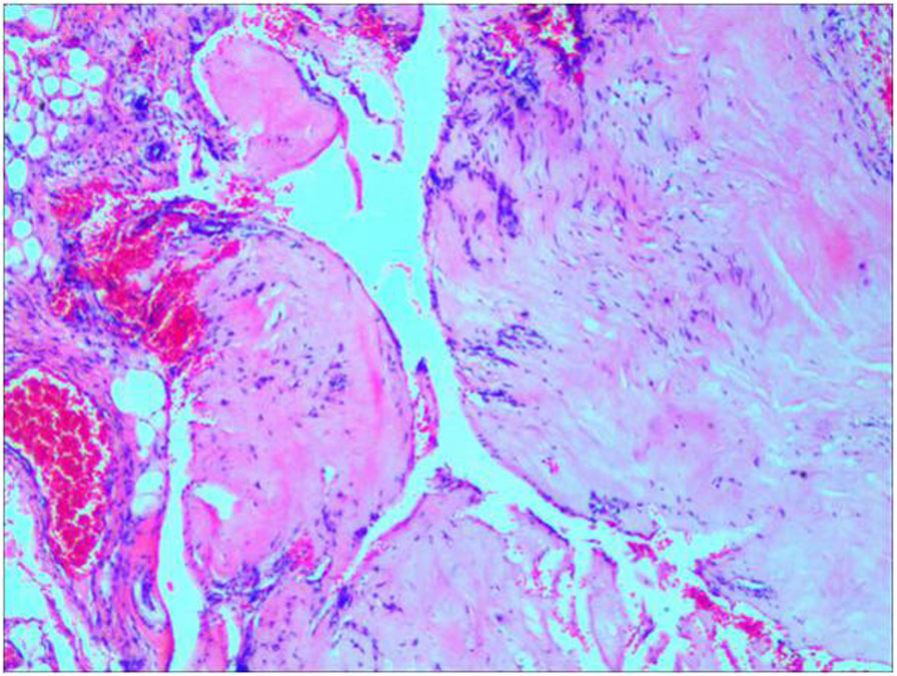
The histopathological diagnosis confirmed that the mass was a degenerated intervertebral disc

A 63-year-old male was admitted to our hospital with back pain and radiating pain in the right lower limb that persisted for 3 months. Upon admission, his neurological examination revealed hypoesthesia of L3 and L4 in the right lower limb. Meanwhile, the patient presented a score of 6 on the VAS and a score of 64 on ODI. A negative Lasegue’s sign was observed on the bilateral limbs. T2-weighted images (T2WI) in the axial planes revealed a dumbbell mass in the lateral recess (Fig. 4b). Gd MRI demonstrated a heterogeneous ring-like enhancement at the periphery of the mass (Fig. 4c). We suspected neurinoma and performed 3D MRI to confirm the diagnosis (Fig. 5). As in case 1, a clear boundary was detected between the mass and nerve root using 3D MRI (Fig. 5). Moreover, the mass was also separated with dura (Fig. 5). Minimally invasive endoscopic surgery was executed in the case (Fig. 6). The histopathological diagnosis confirmed that the mass was a degenerated intervertebral disc (Fig. 7). VAS and ODI score was 1 and 12 at 1 week postoperative follow-up, and 0 and 4 at 1 year postoperative follow-up. The VAS and ODI scores improved from preoperative levels, and there was no occurrence of postoperative complications. The patient remains symptom free at 1-year post-surgery.

## MRI scan

Sequence development was conducted on a 3.0 T whole-body scanner (GE Medical Systems, UK) with a spine coil. Conventional axial T2 fast spin echo (4180/102) (repetition time ms/echo time ms), sagittal T2 fast spin echo (2200/120), and sagittal T1 fast spin echo (460/20) were used to acquire the original images. Then, 3D coronal CUBE-MRN sequence was acquired using the following acquisition parameters for a 1 mm-thick section without an overlapping section gap: a repetition time of 2000 ms, echo time of 32 ms, 352 × 256 matrix, 32 cm file of view, 20°flip angle, and 2 signal-intensity acquisitions. Original coronal fast spin echo images were acquired. We set the imaging plane to be parallel to the longitudinal axis of the lumbar spinal cord and centered on the level of the L3 vertebral body. The coronal image was reconstructed using maximum intensity projection and volume rendering technique on ADW version 4.6 software.

## Discussion

Disc sequestration is defined as the migration of a herniated disc fragment into the epidural space such that it is completely separated from the parent disc [1]. Far migration of disc fragments and the variability in radiological appearance often result in diagnostic confusion prior to operation. A series of reports indicated that some sequestered disc fragments still can be misdiagnosed as other more common epidural and intradural neoplasms, even if CT, MRI, and Gd MRI have been performed before operation [1–19]. For example, Emamian et al. [6] reported one case in which a lumbar herniated disk mimicked a neurinoma in 1993. Subsequently, Sharma et al. [4], Ashkenazi et al. [8], and Levene et al. [5] also demonstrated three cases of disc sequestration that mimicked a nerve sheath tumor. Generally, disc fragments that mimic tumors appear hypointense on T1-weighted images and hyperintense on T2-weighted images. Following contrast medium administration, the patients in these cases presented with ring-like peripheral Gd enhancement. Although the ring-like peripheral enhancement is helpful for differentiating between disc fragments that mimic a tumor and metastatic tumors, hematomas, and meningioma, it is not specific. For example, ring-like peripheral enhancement can also be observed in neurinoma [4–8].

Like the previous reports, we were also unable to make a definitive diagnosis for the present cases depending on CT, MRI, and gadolinium-enhanced MRI before operation because of uncharacteristic radiological appearance. However, within this study we have demonstrated that the use of the coronal MR images of 3D fast-field echo with water selective excitation (3D MRI) was capable to differentiating a sequestered disc fragment from other neoplasms prior to operation.

A series of literatures [1–22] with respect to mimicking tumor disc have been published from 1993 to 2016. Open surgery was performed in all cases [1–22] since the surgeons were unable to make a definitive diagnosis depending on computed tomography (CT), MRI, and gadolinium-enhanced MRI before operation. If the disc fragment of mimicking tumor can be identified prior to operation, open surgery may not be necessary for all patients. We were demonstrated that minimally invasive endoscopic surgery is an alternative, less invasive option to open surgery.

In 2017, Takano et al. [22] successfully identified one patient with disc of mimicking tumor through discography and disco-CT. In this previous study, MRI showed a mass located posterolaterally to the left aspect of the dural sac at the L3 level in a 78-year-old man. The initial diagnosis indicated posterior epidural migration of lumbar disc fragments, malignancy, spontaneous hematoma, or epidural abscess. L3/4 discography clearly showed leakage of the contrast medium into the posterior dural space. Based on these findings, they diagnosed the patients with a neurological deficit due to posterior epidural migration of the lumbar disc fragments. The lesion was identified intraoperatively as a herniated-disc fragment. However, as an invasive technique, complications associated with discography are relatively common and include nerve root injury.

Unlike discography, 3D MRI is a non–invasive imaging examination modality. Compared to conventional MRI, 3D MRI can display the whole view of the nerve root, including the extraforaminal region [23]. Meanwhile, the 3D space location between the herniated disc and nerve root can also be clearly visualized. Although 3D MRI [24, 25] has been used to diagnose symptomatic extraforaminal disc herniation, spinal cord and nerve root compression in recent years, this is the first investigation to indicate that 3D MRI is a helpful imaging tool for differentiating between diagnosis of disc sequestration that mimics a tumor and neurinoma prior to operation.

Since the disc fragment appeared similar to a neurinoma on T1WI and T2WI of MRI in the present cases, neurinoma was speculated prior to operation by us. However, 3D MRI showed a clear boundary between the mass and nerve root. Moreover, the authors found that the mass was also completely separated from the dura in the 3D MRI view. As a result, we excluded neurinoma as a possible diagnosis prior to operation, and minimally invasive endoscopic surgery was performed in the patient. Postoperatively, histopathology confirmed that the mass was consistent with a degenerated intervertebral disc.

## Conclusion

This case report indicates that 3D MRI as a non–invasive imaging examination is a helpful tool for differentiating between an epidural disc fragment that mimicked a tumor and neurinoma prior to operation. If the disc fragment of mimicking tumor can be identified prior to operation, open surgery may not be necessary for all patients. Further verification for the usefulness and deficiency of 3D MRI on differentiating the disc of mimicking tumor ought to be considered using larger sample size in future study.

## Abbreviations

3D MRI: The coronal MRI of 3D fast-field echo with water selective excitation
CT: Computed tomography
MRI: Magnetic resonance imaging
ODI: Oswestry disability index
T1WI: T1-weighted images
T2WI: T2-weighted images
VAS: Visual analog scale
